# Psychological Impact of Inflammatory Bowel Disease on University Students: A Systematic Review

**DOI:** 10.7759/cureus.59176

**Published:** 2024-04-27

**Authors:** Anna Giga, Despoina Pappa, Panagiota Manthou, Maria Chryssi, Thomai Kollia, Despoina Varvitsioti, Emmanouil Giatromanolakis, Nikolaos Anastasiou, Eleni Zigkiri, Polyxeni Mangoulia

**Affiliations:** 1 Nursing, University of West Attica, Athens, GRC; 2 Cath Lab, Henry Dunant Hospital Center, Athens, GRC; 3 Infection Preventionist, University of West Attica, Athens, GRC; 4 Nursing, Saint Savvas, Athens, GRC; 5 Intensive Care Unit, Sotiria General Hospital, Athens, GRC; 6 Nursing, Sotiria General Hospital, Athens, GRC; 7 Medicine, Medical Center of Peristeri, Athens, GRC; 8 Intensive Care Unit, Central Clinic, Athens, GRC; 9 Faculty of Medicine, University of Thessaly, Larissa, GRC; 10 Nursing, University of Athens, Athens, GRC

**Keywords:** crohn’s disease, ulcerative colitis, stress, students, university, ibd

## Abstract

Entering and acclimatizing to a university is crucial for achieving academic goals and graduation. Chronic illnesses can reduce a person’s capacity to perform tasks, whether physically, cognitively, or emotionally, about inflammatory bowel disease (IBD), there is a lack of research about the impact of IBD on the daily lives of students. IBD can be seen as having an adverse effect on the life of college students.

The objective of this review was to examine the psychological ramifications, particularly in relation to stress levels, that IBD elicits in the daily lives of students.

The elementary search utilized specific databases, including PubMed, Web of Science, and Google Scholar. The search terms employed were "IBD," "University," "Students," and "Stress." We reviewed 80 papers and selected 25 for their applicability and relevance. The current review includes at least a total of 12 articles.

The following issues arose: 1) adaption to university, 2) managing IBD individually and from the university setting, 3) social impact, and 4) methods of controlling and coping with the IBD.

Students with IBD have a tough time adapting to new situations. Their emotional and social status plays a significant role in this. The proper management and treatment of IBD throughout studies can have a significant impact on student's academic achievement as well as their later lives.

## Introduction and background

Idiopathic inflammatory bowel diseases (IBDs), such as ulcerative colitis and Crohn's disease, significantly affect the mental well-being and overall quality of life of those afflicted with them [1,2]. A study conducted by Kappelman et al. has revealed an increase in the prevalence of ulcerative colitis and Crohn's disease, particularly among children and adolescents [3]. For instance, a recent study indicates a percentage of 30% in ulcerative colitis cases and an increase of 25% in Crohn's disease cases among this demographic over the past decade [4]. According to Kappelman et al., the validation of registration rates for students has not been conducted [3]. Anxiety, which is occasionally linked to stress, is defined by Lazarus et al. as the physiological and psychological reaction to a terrifying circumstance caused by the surrounding environment [5]. Anxiety is defined by the presence of unpleasant emotions, a depressed mood, and difficulties with emotions [6-9]. Exacerbations in IBD can significantly disrupt individuals' everyday routines as symptoms intensify and the necessity for medical supervision and intervention becomes increasingly crucial [10]. Given these circumstances, students with IBD may have challenges when it comes to enrolling in the university and adjusting to the everyday life within it since they need to adapt to the new environment [10]. Multiple studies have provided evidence of the alterations in lifestyle, dietary habits, and stress levels encountered by female students who suffer from chronic diseases. According to Friedlander et al. and Chemers et al., university students with IBD have a higher level of anxiety and worry compared to their healthy peers [11,12]. Among university students diagnosed with IBD, their sustained high academic performance, particularly in advanced courses such as biochemistry and physiology, serves as a notable characteristic despite the challenges posed by their health condition. Studies have shown that individuals diagnosed with chronic conditions such as IBD often face significant challenges in effectively managing their health, especially during flare-ups or periods of disease exacerbation [13]. This struggle is particularly pronounced in young adults transitioning to university life, where the demands of academic studies, social activities, and self-care can intersect, presenting unique challenges in disease management. In contrast, healthy individuals have better rates of success in both their academic pursuits and professional growth [11-13].

The aim of this review was to examine the psychological ramifications, specifically in relation to stress levels, that IBD elicits in the daily lives of students.

## Review

Materials and methods

This systematic review was conducted using three databases: PubMed, Google Scholar, and Web of Science. The search terms encompassed ulcerative colitis, Crohn's disease, inflammatory bowel disease, stress, daily living, and students. The search was conducted in English. Evaluation of texts involved assessing their title, abstract, and overall completeness. Duplicate entries were removed during the auditing process. The inclusion criteria focused on studies examining the stress experienced by students with IBD in their daily lives. The majority of the studies, published in English, involved adults. Exclusion criteria included studies on children and adolescents and conference papers.

**Figure 1:**
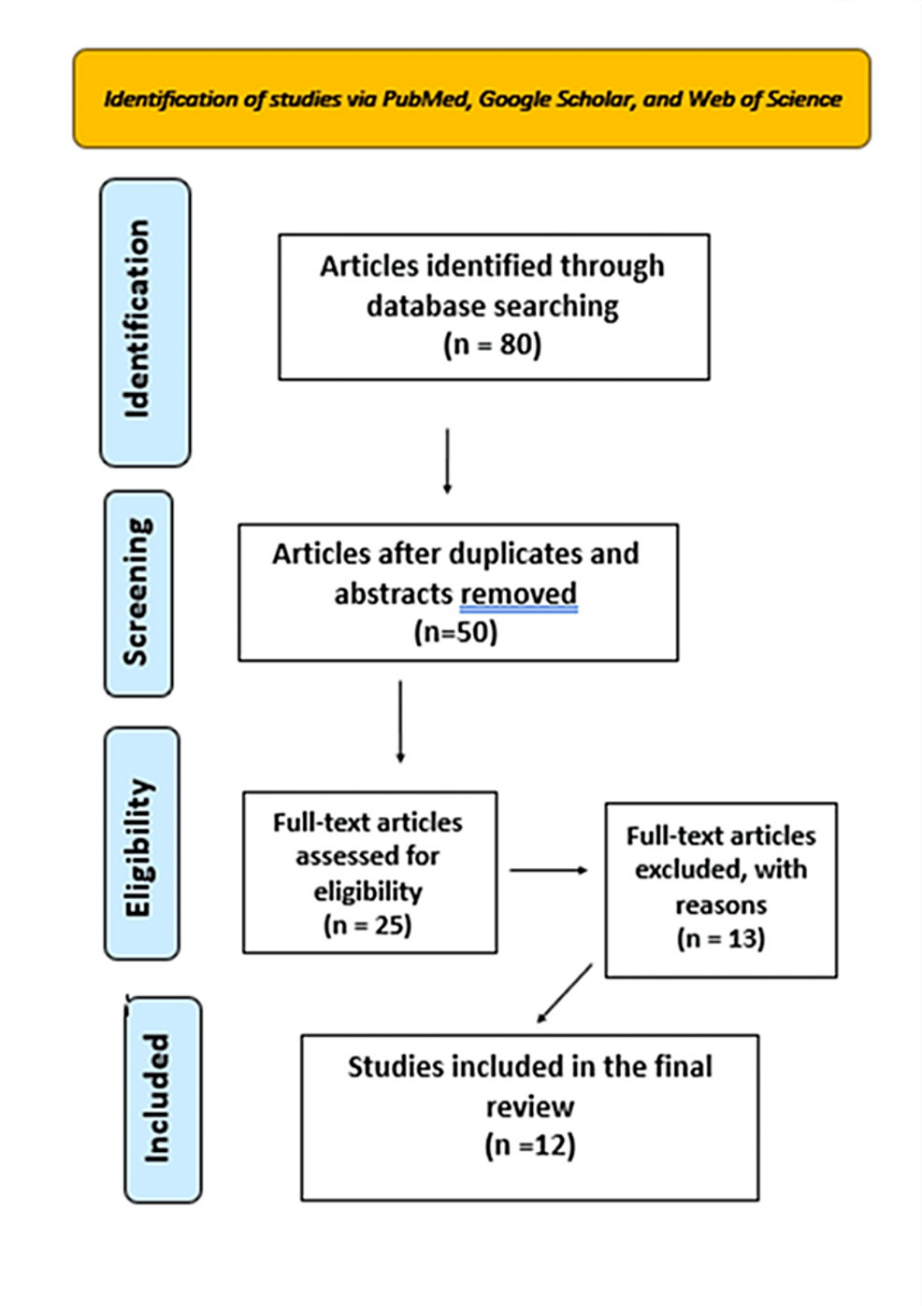
PRISMA flow diagram PRISMA: Preferred Reporting Items for Systematic Reviews and Meta-Analyses

A total of 80 researches has been recorded using electronic searches. After a thorough analysis, duplicate content was removed from 50 publications and abstracts. Twenty-five works were analyzed and assessed for their pertinence to the research subject. A total of 13 published papers were excluded due to their failure to meet the requirements for assessing the psychological impacts on students. Finally, 12 papers were examined for this review (Figure 1).

Specifically, according to the search string, the total report of eighty papers using electronic searches indicates a comprehensive approach to gathering data for the study. This suggests a thorough effort to explore existing literature and studies related to the topic at hand, providing a solid foundation for the subsequent analysis. The process of removing duplicate content demonstrates a commitment to ensuring the quality and integrity of the research. By eliminating redundant information, the analysis can focus more effectively on unique insights and findings from each source. The fact that 25 papers were selected for analysis and assessment out of the total pool of publications and abstracts suggests a discerning approach to choosing relevant literature. This selection process likely involved criteria such as relevance to the research topic, methodological rigor, and quality of evidence.

The decision to exclude 13 publications based on their failure to meet the criteria of assessing psychological impacts on students, highlights the critical importance of upholding rigorous standards in research. This exclusion criterion demonstrates the dedication of this review to maintaining the validity and reliability of their findings. Additionally, the decision not to include studies conducted in languages other than English further reinforces the commitment to ensuring the comprehensiveness and consistency of their analysis.

Results

This review encompasses a grand total of 12 publications, as indicated in Table 1. The worsening of an inflammatory bowel disease significantly affects the patient's psychological well-being. Research has indicated that students who are unwell have elevated levels of concern, remorse, and uncertainty, which negatively impact their overall well-being [14-18]. IBD may have both beneficial and harmful impacts on kids' lives. Garcia-Sanjuan et al. suggests that certain individuals with inflammatory illnesses may adapt and function effectively in their social environments by learning to live with their condition and being accepted by others [19]. 

**Table 1:** Summary table of included papers CD: Crohn's disease, IBD: inflammatory bowel disease, UC: ulcerative colitis, QoL: quality of life

Author	Design	Aim of the study	Sample	Findings
Cohen et al. [1]	Expanded online search	Compare health-related quality of life in CD.	Patients with CD compares with healthy.	Health-related quality of life in patients with CD and health controls.
Agostini et al. [2]	Cross-sectional study	Compare the attachment dimension between IBD patients and healthy control to assess the impact on quality of life in IBD patients.	IBD patients.	Psychological interventions in IBD patients with deterioration in QoL.
Kappelman et al. [3]	Cross-sectional study	Determine the prevalence of CD and UC.	Children with CD/UC.	IBD prevalence has increased over time.
Friedlander et al. [11]	Longitudinal study	Effects of stress, social support, self-esteem.	First-year undergraduate students.	Support self-esteem and mechanisms for stress and social support.
Chemers et al. [12]	Longitudinal study	Effects of academic self-efficacy on academic students.	First-year university students.	Relation between academic self-efficacy and optimism to performance and adjustment.
Bramuzzo et al. [14]	Cross-sectional study	Evaluate the parental distress anxiety of patients with IBD.	Parents with IBD children.	Parental association distress.
Jlenova et al. [15]	Cross-sectional study	IBDs in adolescents are chronic medical conditions with an influence on the quality of life of the families.	Adolescents with IBD and healthy.	No difference in parental style of caring for adolescents.
Werner et al. [17]	Cohort study	Assess the mental health of parents of children with IBD, compared with the age, and gender-matched for mental health problems.	Parents of children with active and inactive IBD.	Parents of children with IBD may need professional support when their child is diagnosed with IBD.
García-Sanjuán et al. [19]	Qualitative study	Explore the experience of caregivers living with relatives affected by CD in a context in which the family provides social support.	Family caregivers of people with CD.	Adaptation to the caring experience. Need for knowledge and control of the disease.
Becker et al. [20]	Online survey	Assess the impact of IBD.	IBD patients.	The impact of IBD among IBD patients and their families.
Graff et al. [21]	Cohort study	Relationship between disease type and activity with psychological quality of life.	IBD patients.	Continued impact on quality of life in IBD, when it is inactive in the psychological characteristics.
Adler et al. [22]	Pilot study	Compare results of a standardized college adjustment survey in students with IBD and without IBD.	Students with CD/UC.	Strategies to increase disease control and provide support.

Conversely, in the study conducted by Becker et al., researchers measured the level of difficulty experienced by both patients and their surroundings in accepting the disease [20]. The study identified various challenges, including emotional distress, social stigma, and practical limitations, faced by individuals and their support networks when coming to terms with the illness [20]. The socioeconomic circumstances of students with IBD often result in dietary restrictions and limitations on alcohol consumption. These constraints can affect their access to necessary food and beverages, adding to the stress they experience. Understanding the impact of these restrictions on their psychological well-being is essential. Therefore, examining how socioeconomic factors intersect with dietary constraints is pertinent to the study’s objective of exploring the psychological ramifications of IBD on students. This aspect sheds light on the broader context in which stress levels are influenced in their daily lives. Due to the illness, individuals have restricted social interactions and often have to reveal their condition [21]. Moreover, the impact of IBD on children is crucial to their academic and overall achievements. IBD patients struggle to manage the time demands of their profession and do badly throughout the evaluation period as a result of escalating stress. The assistance and backing of others play a key role in the academic success of individuals with IBD. University institutions play a crucial role in providing assistance to students with IBD. Research suggests that there is a lack of clinics in rural areas and insufficient equipment and training for the treatment of IBD [22].

This passage discusses the psychological and social impacts of IBD on individuals, particularly focusing on students. Here’s a breakdown of the key points: (i) Psychological well-being: The worsening of IBD significantly affects the patient’s psychological well-being. Students who are unwell experience elevated levels of concern, remorse, and uncertainly, which have a negative impact on their overall well-being [23]; (ii) Beneficial and harmful impacts*:* IBD may have both beneficial and harmful impacts on students’ lives. Some individuals with inflammatory illnesses may adapt and function effectively in their social environments by learning to live with their condition and being accepted by others. However, research also indicates the difficulty experienced by both patients and their surroundings in accepting the disease [23]; (iii) Social and dietary constraints: Students with IBD face dietary restrictions and constraints on alcohol consumption due to their socioeconomic circumstances, which affect their access to food and beverages. Additionally, the illness leads to restricted social interactions and often requires individuals to reveal their condition [23]; (iv) Academic impact*:* The impact of IBD on children is crucial to their academic and overall achievements. Ibd patients struggle to manage the time demands of their profession and may perform poorly throughout the evaluation period due to escalating stress [23]; (v) Support and assistance*: *The assistance and backing of others, including university institutions, play a key role in the academic success of individuals with IBD. however, there is a lack of clinics in rural areas and insufficient equipment and training for the treatment of IBD, highlighting challenges in accessing adequate healthcare resources [23].

Overall, the passage emphasizes the multifaceted challenges faced by individuals with IBD, particularly students, encompassing psychological, social, dietary, and academic aspects, as well as the importance of support and access to healthcare resources.

Discussion

The process of acclimating to the collegiate environment signifies a substantial shift for individuals, signifying a critical juncture in both their personal and scholastic growth [23]. The process of adaptation described here can significantly impact the experiences and outcomes of individuals while they are enrolled at the university. Successful adaptation is, on the one hand, linked to the achievement of social and academic objectives, thereby cultivating a feeling of inclusion and fulfillment. On the other hand, inadequate adaptation can be observed in the form of isolation, disinterest, and subpar academic performance [24]. An essential component in comprehending student adaptation is the determinants that impact their capacity to navigate the complexities and obligations of collegiate existence. According to existing research, the presence of a nurturing family environment is vital in furnishing students with the fundamental support and resources required to achieve academic success [25]. Furthermore, by developing self-assurance and fortitude, pupils are endowed with the cognitive resources necessary to confront challenges and surmount barriers. By implementing efficient stress management techniques, students are able to more effectively manage the demands of their academic studies, extracurricular engagements, and social interactions.

Nevertheless, specific individuals encounter distinct obstacles while making the transition to higher education, especially those who are afflicted with chronic diseases such as IBD. The transition to higher education can cause additional challenges for adolescents with IBD in terms of academic and social integration, in addition to worsening pre-existing health issues [26]. The challenges encountered by adolescents who have chronic illnesses during this period of transition are highlighted in research conducted by Lavigne and Faier-Routman [26]. These findings bring attention to the necessity for interventions and targeted support [26]. Furthermore, the influence of tension on student performance cannot be overstated. The research of Pritchard and Wilson underscores the adverse consequences of heightened levels of stress on academic achievements, thereby emphasizing the criticality of incorporating efficacious stress management techniques in higher education environments [27]. Recognizing the widespread occurrence of IBD among young adults is crucial in order to formulate all-encompassing support structures that are customized to meet the unique requirements of those afflicted [27]. In their study, Lopez-Cortes et al. provided a comprehensive analysis of the psychological intricacies that are linked to IBD, specifically ulcerative colitis and Crohn’s disease [28]. These conditions have the potential to significantly impair the mental and emotional health of affected individuals [28]. Students who are afflicted with IBD frequently face a multitude of obstacles, which encompass challenges with focus, symptom management, and social interaction. IBD has a profound influence that transcends the physical domain, affecting numerous facets of students' everyday existence and presenting substantial obstacles to achieving academic excellence. Although the difficulties encountered by students with IBD are becoming increasingly acknowledged, there is still a scarcity of research that investigates the psychological repercussions for this group [28]. Larsson et al. emphasized the necessity for additional research to be conducted on the psychological aspects and support services that cater to the distinct requirements of this susceptible demographic [29]. By enhancing our comprehension of the convergence of chronic illness and student adaptation, we can more effectively support the scholastic and personal achievements of university-dwelling individuals afflicted with IBD [29].

Studying the psychological impact of IBD on students’ lives is crucial for several reasons. Firstly, IBD symptoms such as pain, diarrhea, and fatigue can significantly disrupt students’ ability to attend classes regularly, concentrate on their studies, and complete assignments on time. Understanding how IBD affects students’ cognitive function and academic performance can help educators and school administrators provide appropriate support and accommodations to help them succeed academically [30].

Also, living with a chronic illness like IBD can take a toll on students’ mental health, leading to feelings of anxiety, depression, and stress. These psychological factors can exacerbate IBD symptoms and reduce students’ overall quality of life. By studying the psychological impact of IBD, healthcare professionals and mental health specialists can develop tailored interventions to address students’ emotional well-being and improve their coping strategies [28].

Ibd symptoms may restrict students’ participation in social activities, leading to feelings of isolation and loneliness. Researching the psychological aspects of IBD can shed light on how the disease affects students’ social relationships, self-esteem, and sense of belonging. This understanding can inform the development of support networks within educational institutions and community settings to help students connect with peers facing similar challenges [29].

Psychological factors such as stress and depression can influence patients’ adherence to medical treatments and dietary restrictions recommended for managing IBD. By examining the psychological impact of IBD on students, healthcare providers can identify barriers to treatment adherence and implement strategies to enhance patients’ motivation and engagement in their healthcare regimen [28].

Ultimately, studying the psychological impact of IBD on students contributes to improving their overall quality of life. By addressing the emotional and social aspects of living with IBD, healthcare professionals, and educators can empower students to navigate their academic and personal lives more effectively, fostering resilience and well-being despite the challenges posed by the disease [30]. In addition, understanding the psychological impact of IBD on students’ lives is essential for providing holistic support and care that addresses not only the physical symptoms of the disease but also the emotional and social aspects that influence their well-being and academic success.

## Conclusions

Inflammatory bowel disease deeply impacts both mental and social well-being of those affected. While the physical symptoms like chronic pain, fatigue and gastrointestinal distress are well-recognized, emerging evidence indicates significant psychological ramifications as well. Despite this growing recognition, research into the psychological effects of IBD, especially among students, is lacking. Surprisingly, few studies have specifically investigated these effects within the student population, leading to a notable gap in understanding how IBD influences mental health, academic performance and social interactions among students grappling with the condition. Addressing this gap is crucial, as it holds the potential to uncover valuable insights into the unique challenges faced by students with IBD and inform the development of targeted interventions and support services aimed at improving their overall well-being and academic success. By prioritizing further research in this area, we can enhance our understanding of the complex interplay between IBD and mental health ultimately leading to more effective interventions and support systems tailored to the needs of students living with IBD.
